# Triterpenoids from *Ocimum labiatum* Activates Latent HIV-1 Expression In Vitro: Potential for Use in Adjuvant Therapy

**DOI:** 10.3390/molecules22101703

**Published:** 2017-10-13

**Authors:** Petrina Kapewangolo, Justin J. Omolo, Pascaline Fonteh, Martha Kandawa-Schulz, Debra Meyer

**Affiliations:** 1 Department of Biochemistry, Faculty of Natural and Agricultural Sciences, University of Pretoria, Hatfield Campus, Pretoria 0002, South Africa; pkapewangolo@unam.na (P.K.); pascaline.fru-fonteh@wits.ac.za (P.F.); 2 Department of Chemistry and Biochemistry, Faculty of Science, University of Namibia, P/Bag 13301, Windhoek 9000, Namibia; kschulz@unam.na; 3 Department of Traditional Medicine, National Institute for Medical Research, P.O. Box 9653, Dar es Salaam 2448, Tanzania; jusiomolo@yahoo.com; 4 Department of Surgery, Faculty of Health Sciences, University of Witwatersrand, 7 York Road, Parktown, Johannesburg 2193, South Africa; 5 Department of Biochemistry, Faculty of Science, University of Johannesburg, Auckland Park, Johannesburg 2006, South Africa

**Keywords:** *Ocimum labiatum*, novel triterpenoids, latent HIV-1, HIV-1 eradication, histone deacetylase, protein kinase C, cytokines

## Abstract

Latent HIV reservoirs in infected individuals prevent current treatment from eradicating infection. Treatment strategies against latency involve adjuvants for viral reactivation which exposes viral particles to antiretroviral drugs. In this study, the effect of novel triterpenoids isolated from *Ocimum labiatum* on HIV-1 expression was measured through HIV-1 p24 antigen capture in the U1 latency model of HIV-1 infection and in peripheral blood mononuclear cells (PBMCs) of infected patients on combination antiretroviral therapy (cART). The mechanism of viral reactivation was determined through the compound’s effect on cytokine production, histone deacetylase (HDAC) inhibition, and protein kinase C (PKC) activation. Cytotoxicity of the triterpenoids was determined using a tetrazolium dye and flow cytometry. The isolated triterpene isomers, 3-hydroxy-4,6*a*,6*b*,11,12,14*b*-hexamethyl-1,2,3,4,6,6*a*,6*b*,7,8,8*a*,9,10,11,12,12*a*,14,14*a*,14*b*-octadecahydropicene-4,8*a*-dicarboxylic acid (HHODC), significantly (*p* < 0.05) induced HIV-1 expression in a dose-dependent manner in U1 cells at non-cytotoxic concentrations. HHODC also induced viral expression in PBMCs of HIV-1 infected patients on cART. In addition, the compound up-regulated the production of interleukin (IL)-2, IL-6, tumour necrosis factor (TNF)-α, and interferon (IFN)-γ but had no effect on HDAC and PKC activity, suggesting cytokine upregulation as being involved in latency activation. The observed in vitro reactivation of HIV-1 introduces the adjuvant potential of HHODC for the first time here.

## 1. Introduction

The current HIV-1 regimen has the ability to reduce viral load to an undetectable level, however, complete eradication of the virus cannot be achieved due to the inability of cART in affecting latent HIV [1]. Latent HIV-1 has the ability to escape immune surveillance [2,3] and latent viral reservoirs established early on during HIV-1 infection are regarded as major contributors to the development of drug resistant HIV-1 strains [4]. Therapeutic targeting of latent HIV-1 is crucial for viral eradication [5,6,7]. A number of clinical trials were conducted with the aim of targeting latent viral reservoirs and the drugs used in these trials were unable to completely purge the highly stable viral reservoirs [2,8]. Treating latent virus would therefore require viral reactivation. Potential drugs tested in clinical trials for reactivation of latent HIV-1 include panobinostat, romidepsin, and vorinostat [9]. Even though these drugs managed to induce HIV-1 transcription, complete destruction of viral reservoirs could not be achieved [9]. Presently, a myeloma drug, Ixazomib is undergoing clinical trials in order to determine its effect on latent HIV reservoirs [10]. Another promising HIV latency reactivator that is currently being considered for human clinical trials to augment cART is prostratin, a phorbol ester isolated from a tropical plant *Homalanthus nutans* (Euphorbiaceae). Prostratin has the unique ability to block HIV-1 infection and at the same time induce latent proviral expression [11,12]. Bryostratin, a marine macrolide isolated from *Bugula neritina* (Linnaeus), can activate HIV from latency [13,14]. Total synthesis of bryostratin has been achieved and the in vivo safety and efficacy of the synthetic compound is under investigation [15,16,17]. 

The pharmacological effect of novel compounds from nature such as prostratin and bryostratin continue to support the fact that nature remains a great source of novel compounds, which is also why most existing drugs are derived from natural sources [18]. Lamiaceae is a plant family with numerous ethnobotanical uses [19,20]. Plants from this family are rich in terpenoids, a class of compounds commonly associated with the medicinal activity of these plants [21,22]. Various terpenoids reportedly inhibit different stages of the HIV-1 life cycle including in vitro inhibition of viral enzymes like protease [23], and in vivo blocking of viral maturation [24,25,26].

In this study, novel triterpenes isolated from *Ocimum labiatum* (Lamiaceae) were investigated for the potential to activate latent HIV-1 in a chronically infected monocytic U1 cell line. The isolated terpenes activated latent HIV, suggesting possible use of the triterpenes as adjuvants in conjunction with cART. Adjuvant therapy in this context means the use of a compound (in this case a natural product) in concert with cART; so that the former can activate latent virus replication while cART inhibits viral replication and potentially eradicate latent HIV reservoirs. The viral reactivation mechanism was investigated by monitoring the ability of the compounds to inhibit HDAC, activate PKC, or upregulate proinflammatory cytokines linked to latency activation. There is a continuous search for novel compounds to include in future clinical trials for use as adjuvant therapy [8,27] and the present report is a contribution towards that goal.

## 2. Results

### 2.1. Compounds Isolation

**Figure 1:**
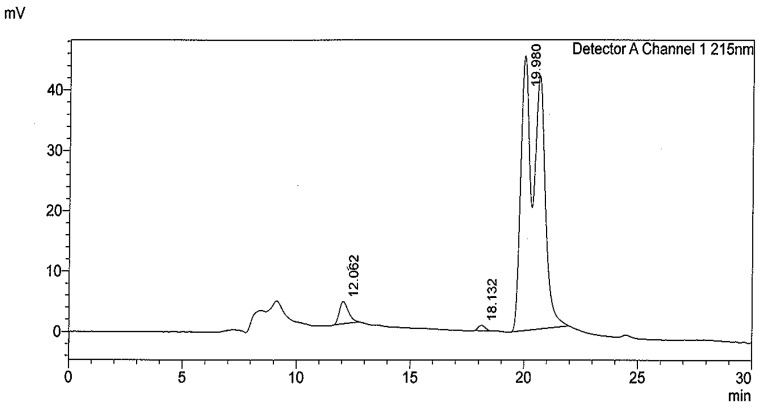
High performance liquid chromatography (HPLC) chromatogram of HHODC isomers.

Column chromatography of the ethyl acetate fraction of *O. labiatum* leaves on silica gel followed by purification with Sephadex LH-20 led to the isolation of two triterpenes. A known triterpene that is common in nature, amyrin [28] was identified as compound **1** while compound **2**, the second triterpene,3-hydroxy-4,6*a*,6*b*,11,12,14*b*-hexamethyl-1,2,3,4,6,6*a*,6*b*,7,8,8*a*,9,10,11,12,12*a*,14,14*a*,14*b*-octadecahydropicene-4,8*a*-dicarboxylic acid isolated appeared as a mixture of isomers. HPLC analyses of HHODC showed two closely adjacent peaks on the HPLC chromatogram (Figure 1) which is typical of isomers [29]. Peaks 1 and 2 had the same retention time.

### 2.2. Viability Studies

The CC_50_ value of HHODC in U1 cells was 8.2 ± 0.1 µg/mL while amyrin demonstrated low toxicity in U1 cells with a CC_50_ of > 100 µg/mL. The CC_50_ value for auranofin, the toxic compound used as control for cytotoxicity, was < 10 µM.

### 2.3. Effect of HHODC on Viral Expression

**Figure 2:**
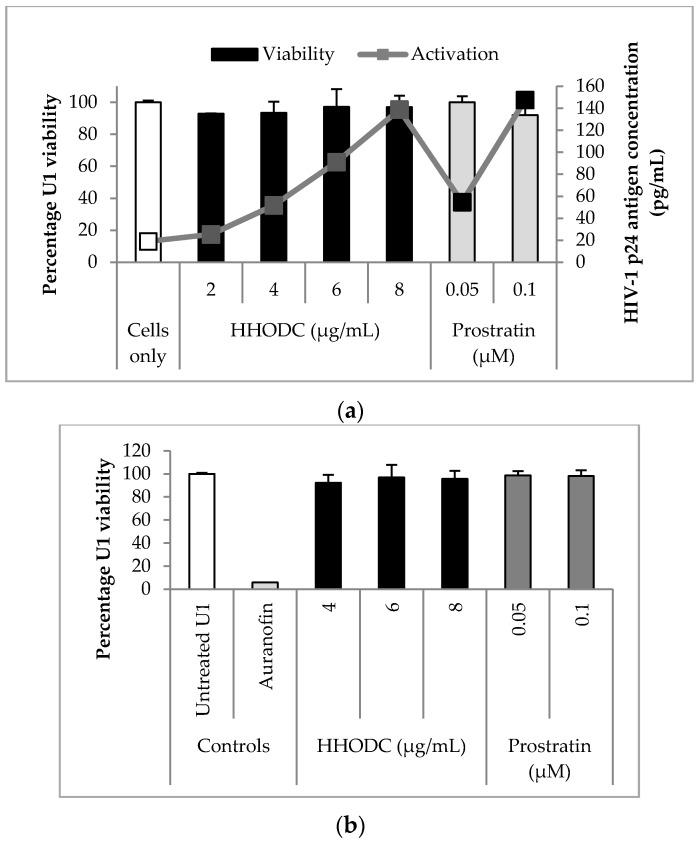
Effects of compounds on HIV-1 expression and U1 proliferation. U1 cells were treated with 2, 4, 6, and 8 µg/mL HHODC. Each value is expressed as mean ± standard deviation (*n* = 3). (**a**) Supernatant was collected after 72 h incubation and quantitatively analysed for HIV-1 p24 antigen. HHODC activated latent HIV-1 expression in a dose-dependent manner. Prostratin (0.1 and 0.05 µM) was included as a positive control of latent HIV-1 activation in U1 cells, while the toxic compound, auranofin (10 µM) was included as positive control for U1 viability. MTT assay revealed U1 viability of >80% at the concentrations tested for HIV-1 expression; (**b**) Flow cytometry was used to confirm viability observed with MTT. U1 cells labelled with CFSE were treated with active concentrations of HHODC and prostratin for 72 h. PI was included to exclude dead cells. Each bar reflects the mean of 2 independent experiments ± SD, analysed in triplicates.

HHODC induced HIV-1 expression from U1 cells in a dose-dependent manner (Figure 2a), with concentrations of 2, 4, 6, and 8 µg/mL increasing viral p24 levels by 1.3, 2.7, 4.8, and 7.3 folds, respectively. Amyrin did not significantly (*p* < 0.05) induce HIV-1 expression in U1 cells (Figure S1). Unstimulated U1 cells were characterized by a state of relative latency and low detectable HIV-1 p24 antigen. Prostratin was used as a known latency activator and reactivated latent HIV-1 by 2.9 and 7.8 folds at 0.05 and 0.1 µM respectively. The concentrations of HHODC and prostration tested for HIV-1 expression were not cytotoxic to U1 cells as shown in a concurrent MTT study where viability for both compounds was greater than 80% (Figure 2a). Flow cytometric analysis using CFSE [30] was used to confirm concentrations of HHODC that appeared non-cytotoxic with MTT viability studies (Figure 2b). Perhaps it is worth noting here that HHODC was tested for inhibitory activity against HIV-1 protease but did not appreciably inhibit the enzyme (Figure S2).

### 2.4. Effect of HHODC on Natural Infection

**Figure 3:**
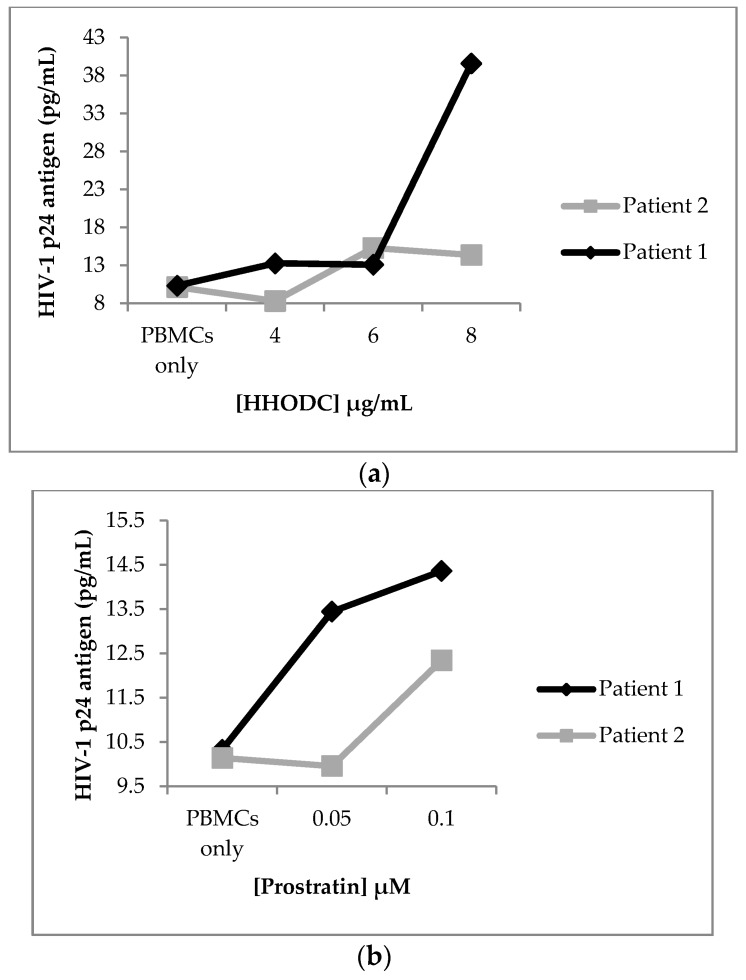
HHODC induces HIV-1 expression from PBMCs of infected patients on cART. (**a**) PBMCs from cART patients were treated with HHODC and (**b**) prostratin as indicated. Supernatant was collected after 72 h to determine HIV-1 p24 antigen by ELISA. Each point represents the mean of 2 independent experiments ± SD, analysed in triplicates.

**Figure 4:**
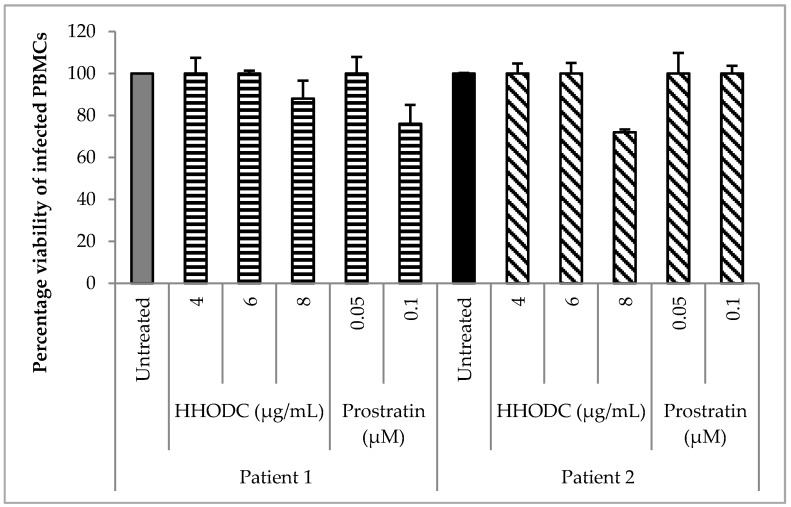
HHODC and prostratin’s effect on infected PBMC viability. Concentrations of the two compounds that activated viral latency resulted in PBMC viability of > 70% for both patients. Each bar represents the mean of 2 independent experiments ± SD, analysed in triplicates.

HHODC had an effect on HIV-1 expression in cells isolated from 2 HIV-infected patients on cART as illustrated in Figure 3a. PBMCs from patient 1 elicited the most response for viral expression after treatment with 8 µg/mL of HHODC. There was also an increase in HIV-1 p24 concentration in PBMCs from patient 2, most notably at the two highest concentrations tested for HHODC (6 and 8 µg/mL). Treatment with prostratin resulted in minimal increases in p24 antigen production (Figure 3b). The concentrations tested for HHODC demonstrated minimal cytotoxicity on PBMCs with cell viability greater than 70% (Figure 4). HIV-1 p24 antigen concentration was compared to that in an untreated PBMCs control (PBMCs only).

### 2.5. Viral Reactivation Mechanism of HHODC

HHODC demonstrated marginal HDAC inhibition at concentrations of 6, 8, and 12.5 µg/mL (Figure S3a). At 6 and 8 µg/mL, HHODC only minimally activated PKC from cell lysates when compared to prostratin (Figure S3b). These findings suggest that neither HDAC inhibition nor PKC activation were important in the viral reactivation caused by HHODC, but both pathways could potentially be contributing to the observed viral reactivation. 

### 2.6. Effect of HHODC on the Endogenous Production of Pro-Inflammatory Cytokines

The concentration of HHODC tested for cytokine production was 6.3 µg/mL; the same concentration that activated latent HIV-1 in U1 cells. HHODC significantly (*p* < 0.05) increased the production of IL-2, IL-6, TNF-α, and IFN-γ in PBMCs, compared to untreated controls (Table 1). Similar, although not significant, up-regulation (except in the case of IFN-γ) was observed in U1 cells following HHODC treatment. As expected, the cell mixture (PBMCs) produced higher cytokine difference compared to the U1 cell line in which cytokine treatment is usually applied to regulate viral expression. Latency reactivators usually upregulate the production of IL-6 and TNF-α which is in line with observations presented here.

A concurrent study on the viability of the PBMCs was confirmed to be 100% after treatment with 6.3 µg/mL of HHODC (Figure S4).

## 3. Discussion

Current HIV drugs target different stages of the viral life cycle, however, none of the drugs in the regimens target latent HIV-1 reservoirs [31]. These reservoirs can replenish systemic infection when treatment is interrupted and also contribute to the development of drug resistant HIV-1 strains [32]. Ideal antiretroviral therapy should include drugs with the ability to activate latent HIV-1 reservoirs in order for HIV inhibition drugs in the regimen to interrupt the active replication with the goal of eventually eradicating the virus. 

Here, a triterpene isolated from *O. labiatum* (HHODC) for the first time was able to activate latent HIV-1 and the viral expression was comparable to that induced by prostratin. The activation occurred at a non-cytotoxic concentration suggesting that HHODC has potential as an inductive adjuvant for cART. HHODC also induced viral expression in primary cells isolated from HIV-1 infected patients on cART, suggesting potential clinical applicability. PBMCs from infected individuals on cART was deliberately used in this study as the reputation of cART in lowering viral load but not eliminating viral reservoirs would support latency reactivation when increased p24 levels were detected in the presence of HHODC.

Amyrin, a commonly occurring triterpene in nature [28], was also isolated from *O. labiatum* leaves in the course of this investigation. It is the first time amyrin was isolated from this plant. Amyrin did not demonstrate HIV activation/inhibition potential when tested in the p24 ELISA assay. It is, however, a valuable compound since it has been reported to demonstrate hepatoprotective effects [33].

The viral reactivation approach is regarded as one of the major strategies for purging latently-infected cells. There are various mechanisms by which latent HIV can be activated. Prostratin and bryostratin, compounds from natural sources, induce HIV expression in latent cells by activating PKC [14]. PKC can be modulated by small molecular agents to induce the expression of latent HIV-1 from infected cellular reservoirs [34]. Inhibiting HDACs is another mechanism by which latent HIV can be reactivated [14]. HDACs produce hypoacetylated nucleosomes at the HIV promoter region and this reduces access to transcription factors which contributes to the maintenance of HIV latency [35]. Therefore, HDAC inhibitors reactivate HIV from latency [36] by allowing access to transcription factors required for viral expression. A number of compounds have been identified as potential HDAC inhibitors with some examples being romidepsin, vorinostat, and panobinostat [14,37,38]. The triterpenoid investigated in this study, HHODC, did not significantly inhibit HDAC nor activate PKC. While the minimal inhibition and activation of HDAC and PKC respectively could have been contributing to the reactivation of virus, it is possible that the observed latent HIV activation was through one of many other mechanisms by which compounds can reactivate the virus [14]. Alternatively, it could have been through a completely novel mechanism since the structure of HHDOC differs from that of other latent virus activators. 

Further exploration of the mechanism by which HHDOC activated HIV from latency was through investigating its effect on the endogenous production of the pro-inflammatory cytokines, IL-2, IL-6, TNF-α, and IFN-γ. Even though cytokines were not expressed at similar concentration levels in PBMCs and U1 cells, upregulation of cytokines was mostly observed. The lesser upregulation of cytokines in U1 cells follow a similar pattern as what was observed in PBMCs, but on a smaller scale. The minute downregulation of IFN-γ in U1 cells compared to its significant upregulation in PBMCs could be because IFN-γ is primarily produced by natural killer and natural killer T cells, and by CD4 Th1 and CD8 cytotoxic T lymphocyte effector T cells all of which are found in PBMCs [39,40,41], while in the established promonocytic U1 cell line, its arrested developmental state limits the production of cytokines.

Activation of TNF-α is a non-specific mechanism of viral reactivation which has been reported for prostratin and PMA [11,42]. HHODC could have possibly induced viral expression by modulating an array of cytokines in U1 cells; cytokines implicated in the stimulation of latent HIV-1. Cytokines affected by HHODC in this study, IL-2, IL-6, TNF-α, and IFN-γ, are documented as being among the many biomarkers affected by HIV-1 infection due to immune dysfunction [43,44,45]. IL-6 is reportedly elevated during HIV infection and has been associated with mortality and opportunistic infections [43,46]. Worsley et al [44] reported an increase in IFN-γ levels in a group of HIV-infected individuals with immune reconstitution inflammatory syndrome. IL-2 was used in early clinical attempts to purge latent HIV-1 in vivo where the cytokine had a significant effect on latent cellular reservoirs, but viral rebound was still observed when antiretroviral therapy was discontinued [14].

A study by Poli et al [47] reported the ability of IL-6 to synergistically induce the production of HIV-1 expression in U1 cells. IL-6 synergized with IL-1 in the upregulation of virus expression in the cells [47]. In another study, IFN-γ was found to be a potent modulator of HIV-1 expression; direct stimulation of U1 cells with IFN-γ activated HIV-1 in U1 cells suggesting this cytokine to play an important role as an inducer of latent HIV-1 [48]. In the present study, the ability of HHODC to up-regulate IL-2, IL-6, TNF, and IFN-γ in monocytic cells suggests a possible viral inducing mechanism of the compound which could be through the up-regulation of the aforementioned cytokines.

A number of studies reported the expression of IL-1, TNF-α, and nuclear factor kappa B (NF-κβ) in PMA-treated U1 cells [47,49,50]. These studies confirm the ability of U1 cells to produce cytokines, supporting the data presented here. U1 cells were derived from a promonocytic cell line, U937, which has been reported to express various cytokines [51], further supporting the possibility of cytokine production from these cells when stimulated with various agents. In part, this explains the mechanism by which HHODC was able to activate latent HIV-1 in this study; the isomers possibly induced the production of IL-2, IL-6, TNF, and IFN-γ which in turn activated latent HIV-1.

## 4. Materials and Methods 

### 4.1. General

^1^H- and ^13^C-NMR were recorded on Bruker 400 MHz (Bruker, Billerica, MA, USA) NMR spectrometer using tetramethylsilane as internal standard. The solvents used for NMR spectra were deuterated chloroform and deuterated methanol. TLC was carried out on Merck (Merck, Darmstadt, Germany) silica gel plates F_254_ with layer thickness of 0.2 mm and visualized under UV light and by staining with vanillin-sulphuric acid, followed by heating. Column chromatography separation and purification were performed on silica gel 60: 70–230 mesh (Merck, Darmstadt, Germany) and Sephadex LH-20 (Sigma, St. Louis, MO, USA). HPLC was conducted with a Shimadzu preparative 6AD LC system equipped with a UV-visible (215 and 254 nm) detector, a manual injector 10AF, and a fraction collector FRC-10A (Shimadzu, Kyoto, Japan). Aliquots (200 µL) were injected in a C18 Jupiter analytical column of 250 mm × 4.6 mm × 10 µm (particle size).

### 4.2. Plant Material

Fresh leaves of *Ocimum labiatum* were collected in February 2012 from the Botanical Garden of the University of Pretoria. A voucher specimen is deposited in the H.G.W.J Schweikerdt Herbarium of the University (117693).

### 4.3. Isolation and Identification of Compounds

**Figure 5:**
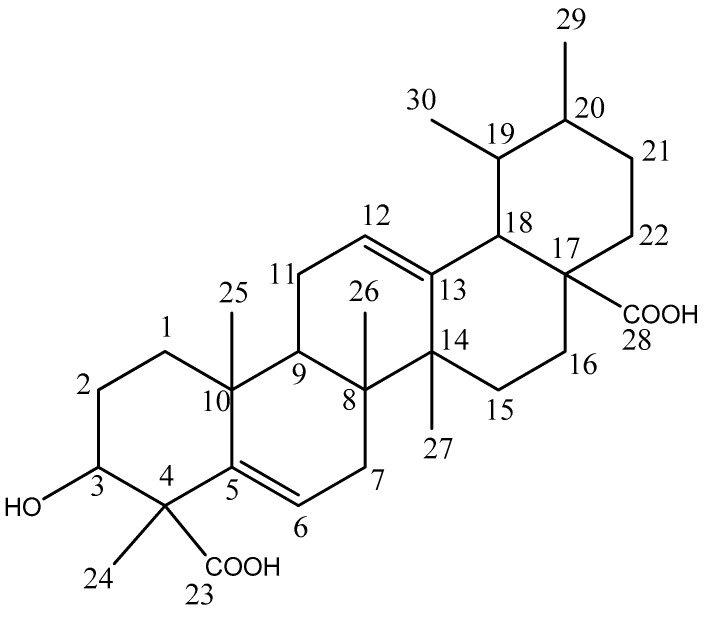
New triterpenoid compound isolated from *Ocimum labiatum*.

Fresh leaves (894.6 g) were extracted in ethanol. The obtained filtrate was concentrated under vacuum and the residue was re-dissolved in ethyl acetate to exclude highly polar tannin compounds [52]. The ethyl acetate fraction was partitioned on silica gel with *n*-hexane–ethyl acetate (100:0 to 0:100) successively to afford fractions which were further purified with Sephadex LH-20 eluted with chloroform. Separation over a Sephadex column yielded compounds **1** (24.9 mg) and **2** (250 mg). The spectral data for compound **1** were in agreement with information in the literature for amyrin [28,53]. Compound **2** (Figure 5) was identified from the NMR spectra to be a new triterpenoid, 3-hydroxy-4,6*a*,6*b*,11,12,14*b*-hexamethyl-1,2,3,4,6,6*a*,6*b*,7,8,8*a*,9,10,11,12,12*a*,14,14*a*,14*b*-octadecahydropicene-4,8a-dicarboxylic acid (abbreviated to HHODC). The spectral assignments for HHODC are presented in Table 2. Compound **2** or HHODC is the main focus of this study. Compounds were reconstituted in dimethyl sulfoxide (DMSO), which provides a sterile environment, before each biological assay. Further dilutions to obtain desired compound concentrations were done in cell culture media.

### 4.4. Cytotoxicity of Isolated Compounds

The cytotoxic effect of amyrin and HHDOC on U1 cells was measured using 3-(4,5-dimethylthiazol-2-yl)-2,5-diphenyltetrazolium bromide (MTT; Sigma, St. Louis, MO, USA) [54]. Briefly, cells were seeded at 1 × 10^4^ cells/well in 96-well plates and incubated with various concentrations of the compounds for 72 h at 37 °C in a humidified incubator with 5% CO_2_. After the 72 h incubation, MTT solution was added to the cells and incubated for an additional 2 h. The formazan crystals produced by viable cells were dissolved in 50 µL of 1 M hydrochloric acid in propanol, and the absorbance was measured at 550/690 nm using a Multiskan Ascent microplate reader (Thermo Labsystems Inc., Beverly, MA, USA). Auranofin, a toxic compound with anti-tumour activity [55], was used as a positive control for cytotoxicity. A repeat of 6 experiments (*n* = 6) was conducted in triplicates and viability data was reported as CC_50_ values.

### 4.5. Stimulation of Latent HIV-1 Production

#### 4.5.1. HIV-1 Expression from Latently Infected Promonocytic U1 Cells

The latently infected promonocytic U1 cells [50] were obtained from the AIDS Reagent program, Division of AIDS, National Institute of Health (Rockville, MD, USA). In order to obtain an appreciable amount of p24 in the supernatant, U1 cells were seeded at 1 × 10^5^ per well in a 24-well plate and treated with desired concentrations of HHODC and prostratin (Sigma, St. Louis, MO, USA). Prostratin is a known latent HIV-1 inducer [11]. A population of untreated U1 cells was included as a control. All stimulation experiments were performed in triplicate. Supernatants were drawn after 72 h, stored at −20 °C, and the concentration of HIV-1 p24 antigen was analysed using a RETRO-TEK HIV-1 p24 antigen ELISA kit (ZeptoMetrix Corporation, Buffalo, NY, USA). To establish that the induced virus production was caused by the activation of HIV-1 expression and not due to toxicity, a viability dye MTT was added to U1 cells after harvesting supernatant. In a separate replica experiment, U1 cells were labelled with a fluorescent dye (carboxylfluorecein succinimidyl ester; CFSE) prior to treatment with desired concentrations of HHODC, prostratin, and auranofin for 72 h. Auranofin was included as a positive control for cytotoxicity. Propidium iodide (PI) was incorporated in CFSE labelled cells to differentiate dead from viable and proliferating cells [52]. Data was acquired on a FACSAria (BD BioSciences, San Jose, CA, USA).

#### 4.5.2. HIV-1 Expression from PBMCs of Infected Individuals on cART

Ethical approval for obtaining blood samples from consenting donors was granted by the Faculties of Natural and Agricultural Sciences, and Health Sciences Ethics Committees (EC080506-019;163/2008, University of Pretoria, South Africa). Fresh whole blood from two infected patients on cART was treated to density centrifugation on a Ficoll–Hypaque (Sigma, St. Louis, MO, USA) gradient to obtain peripheral blood mononuclear cells (PBMCs). PBMCs were seeded at 5 × 10^5^ per well in a 24-well format and treated with HHODC (4, 6 and 8 µg/mL) and prostratin (0.05 and 0.1 µM). Supernatant was drawn after 72 h and analysed for p24 concentration using a RETRO-TEK HIV-1 p24 antigen ELISA kit (ZeptoMetrix Corporation, Buffalo, NY, USA). The viability of treated cells was evaluated by adding MTT after the removal of supernatant.

### 4.6. Viral Reactivating Mechanism

Reactivation of virus by small molecules occurs through different mechanisms [56]. Here three of such methods were explored.

#### 4.6.1. Effect of HHODC on Histone Deacetylase Activity

HDAC inhibition is one of the mechanisms attributed to the activation of latent HIV-1 in viral reservoirs [57]. A fluorometric HDAC assay kit (Sigma, St. Louis, MO, USA) was used to assess HDAC inhibition by HHODC. The assay provides a simple enzymatic reaction for the detection of HDAC activity using a substituted peptide as a substrate. The peptide has an acetylated lysine residue and a bound fluorescent group. The kit provides a HeLa cell lysate as a source of HDAC activity. The first step of the reaction is deacetylation of the acetylated lysine side chain by HDAC and the second step involves the cleavage of the deacetylated substrate by the developer solution, releasing a free highly fluorescent group. HHODC was incubated with HDAC at various concentrations. Trichostatin A (TSA) was used as a positive control for HDAC inhibition [58]. Other controls included substrate in assay buffer only and an untreated enzyme control. Fluorescence was measured with a spectrofluorometer (Thermo Labsystems, Beverly, MA, USA) at an excitation wavelength of 355 nm and an emission wavelength of 460 nm.

#### 4.6.2. Effect of HHODC on Protein Kinase C Activity

The PKC activity kit obtained from Enzo Life Sciences (Enzo Life Sciences, Inc., Farmingdale, NY, USA) was used for this assay and the protocol followed was according to the manufacturer’s instructions. PKC is another enzyme which is activated by latency activators such as prostratin [11]. PKC activity was measured from cell lysates of U1 cells treated with 6 and 8 µg/mL of HHODC for 72 h (37 °C, 5% CO_2_). Prostratin was used as a positive control for PKC activation.

#### 4.6.3. Effect of HHODC on the Production of Pro-inflammatory Cytokines

Pro-inflammatory cytokines such as IL-2, IL-6, INF-γ, and TNF-α have been shown to activate latent HIV in vitro [14,42,48,50]. HHODC was investigated for its effect on an array of cytokines in HIV negative PBMCs and U1 cells using a Th1/Th2/Th17 cytometric bead array (CBA) technique. CBA enables the quantification of IL-2, IL-4, IL-6, IL-10, TNF-α, IFN-γ, and IL-17A. For the purposes of this study, the main focus was on the endogenous production of TNF-α, INF-γ, IL-2, and IL-6 since up-regulation of these cytokines has been associated with viral reactivation [14,42,48,59].

### 4.7. Statistical Analysis

Data for all experiments is presented as the mean ± SD. Significant differences and CC_50_ values were computed using Graphpad Prism 5 (Graphpad Software Inc., La Jolla, CA, USA) and Student’s t test for unpaired observations. A *p* < 0.05 was considered significant. Flow cytometry data was analysed using FlowJo Version 7.6.1 (TreeStar Inc., Ashland, OR, USA).

## 5. Conclusions

This in vitro study identified a potential inducer of HIV-1 expression, HHODC, from a natural source. The ability of HHODC to activate latent HIV at a non-cytotoxic concentration may present an important contribution to the improvement of therapeutic strategies to control HIV replication by targeting latent viral reservoirs. The data presented here provides promising evidence for the further investigation of HHODC’s development as a potential adjuvant of cART.

## Figures and Tables

**Table 1:** Effect of HHODC on cytokine production in PBMCs and U1 cells (*n* = 2).

	Cytokine Concentrations (pg/mL)			
	PBMCs		U1 Cells	
Analyte	Untreated	Treated with HHODC	Untreated	HHODC
IL-2	21.0 ± 6.4	26.9 ± 6.9	0	2.01 ± 5.4
IL-6	2801.7 ± 21.9	14744.9 ± 17.9	1.4 ± 6.3	1.6 ± 4.6
TNF-α	164.1 ± 26.5	528.7 ± 27.7	1.7 ± 4.4	2.2 ± 4.9
IFN-γ	251.0 ± 22.9	842.6 ± 21.0	2.5 ± 4.6	1.8 ± 4.7

**Table 2:** ^1^H- and ^13^C-NMR data of compound 2 (400 MHz, in CD_3_OD).

Position	^13^C δ ppm	^1^H δ mult
1	36.7 (*t*)	1.41, 2H, (*m*)
2	27.3 (*t*)	1.61, 2H, (*m*)
3	78.3 (*d*)	2.89, 1H (*m*)
4	55.3 (*s*)	
5	143.8 (*s*)	
6	122.2 (*d*)	5.26, 1H, (*m*)
7	33.9 (*t*)	2.02, 2H, (*s*)
8	39.0 (*s*)	
9	47.8 (*d*)	1.54, 1H, (*m*)
10	30.2 (*s*)	
11	26.4 (*t*)	2.15, 2H, (*m*)
12	125.4 (*d*)	5.25, 1H, (*m*)
13	138.2 (*s*)	
14	41.3 (*s*)	
15	27.8 (*t*)	1.35, 2H
16	25.0 (*t*)	1.64, 2H
17	48.1 (*s*)	
18	52.9 (*d*)	2.25, 1H
19	38.6 (*d*)	1.71, 1H
20	38.4 (*d*)	1.67, 1H
21	30.4 (*t*)	1.58, 2H
22	33.5 (*t*)	1.98, 2H
23	180.3 (*s*)	
24	18.1 (*q*)	1.18, 3H (*s*)
25	20.1 (*q*)	1.14, 3H (*s*)
26	14.9 (*q*)	0.93, 3H (*s*)
27	23.9 (*q*)	1.00, 3H (*s*)
28	180.5 (*s*)	
29	23.1 (*q*)	0.80, 3H
30	16.3 (*q*)	0.87, 3H

## Supplementary Materials

Supplementary materials are available online. Figure S1: Effect of amyrin on HIV-1 expression, Figure S2: Effect of HHODC on HIV-1 PR, Figure S3a,b: Effects of HHODC on HDAC and PKC activities, Figure S4: Effect of HHODC on the viability of PBMCs.
