# Supplementary material for: Triterpenoids from Ocimum labiatum Activates Latent HIV-1 Expression In Vitro: Potential for Use in Adjuvant Therapy
Source: Molecules. 2017 Oct 13;22(10):1703. doi: 10.3390/molecules22101703 (PMC6151608; doi:10.3390/molecules22101703)
Supplement: Supplementary file 1 [file molecules-22-01703-s001.pdf]

Figure S1: Effect of amyrin on HIV-1 expression. Amyrin did not significantly ($p<0.05$) induce HIV-1 expression in the latently infected monocytic cell line.

Figure S2: Effect of HHODC on HIV-1 PR (n = 4). The compound, fluorogenic substrate and HIV-1 PR were incubated at 37°C for 1 h. HHODC demonstrated inhibition of <50% (100 μg/mL). AP=Acetyl pepstatin a known protease inhibitor was used as control.

Figure S3: Effects of HHODC on HDAC and PKC activities. (a) HDAC activity in HeLa cell lysate was measured by incubating substrate and samples in a reaction volume 100 μL. Marginal HDAC inhibition was observed with 8 and 12.5 μg/mL of HHODC, while higher HHODC concentrations increased enzyme activity. (b) An insignificant ($p>0.05$) 20% activation was observed at 8 μg/mL and 9% at 6 μg/mL compared to 80.7% for prostratin, the positive control.

Figure S4: Effect of HHODC on the viability of PBMCs. The concentration tested for endogenous cytokine production, 6.3 μg/mL, was not toxic in PBMCs as illustrated. Auranofin was used as a positive control for toxicity. Percent cell viability in the presence of HHODC was 100%.

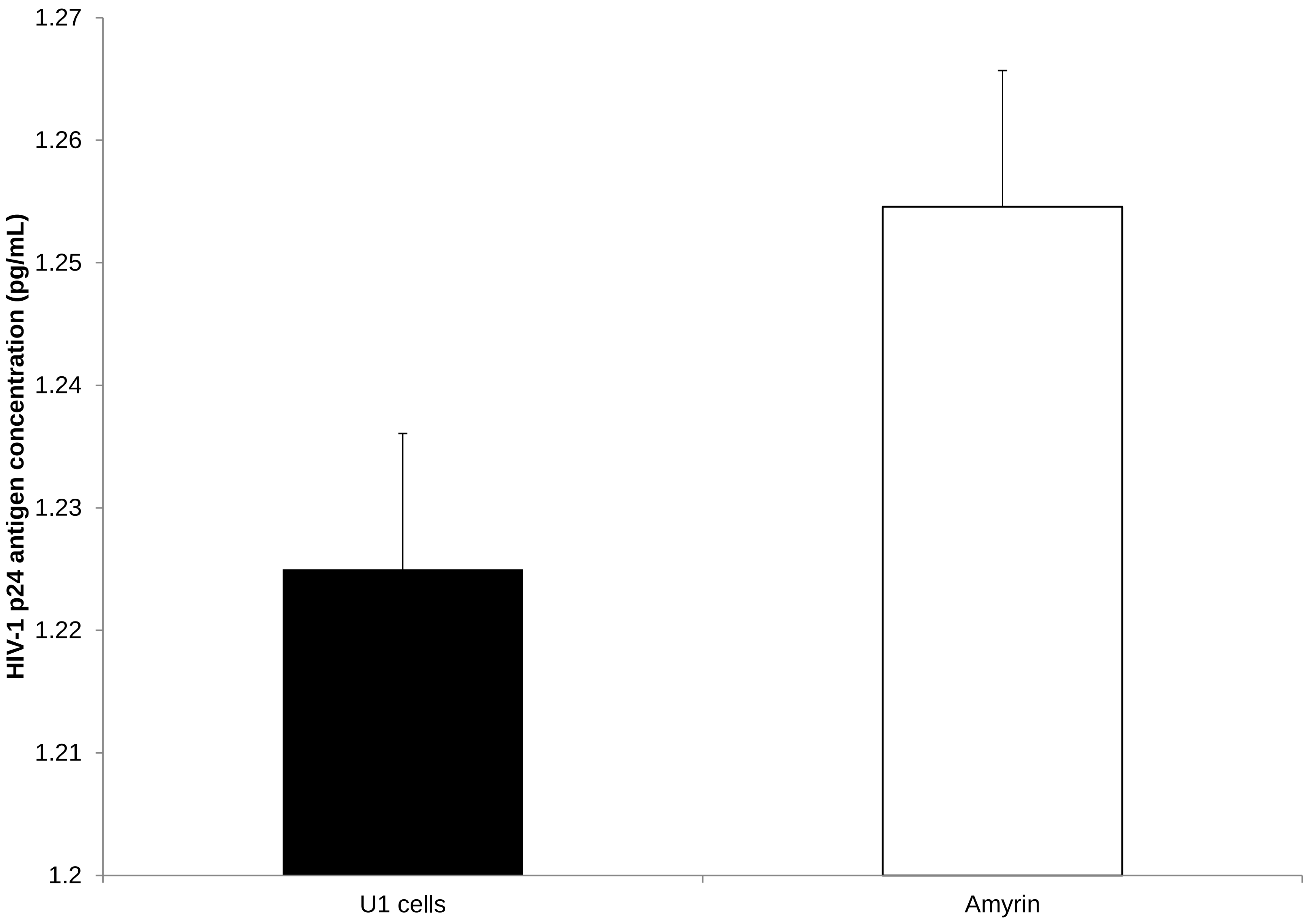

HIV-1 p24 antigen concentration (pg/mL)
1.27
1.26
1.25
1.24
1.23
1.22
1.21
1.2
U1 cells
Amyrin

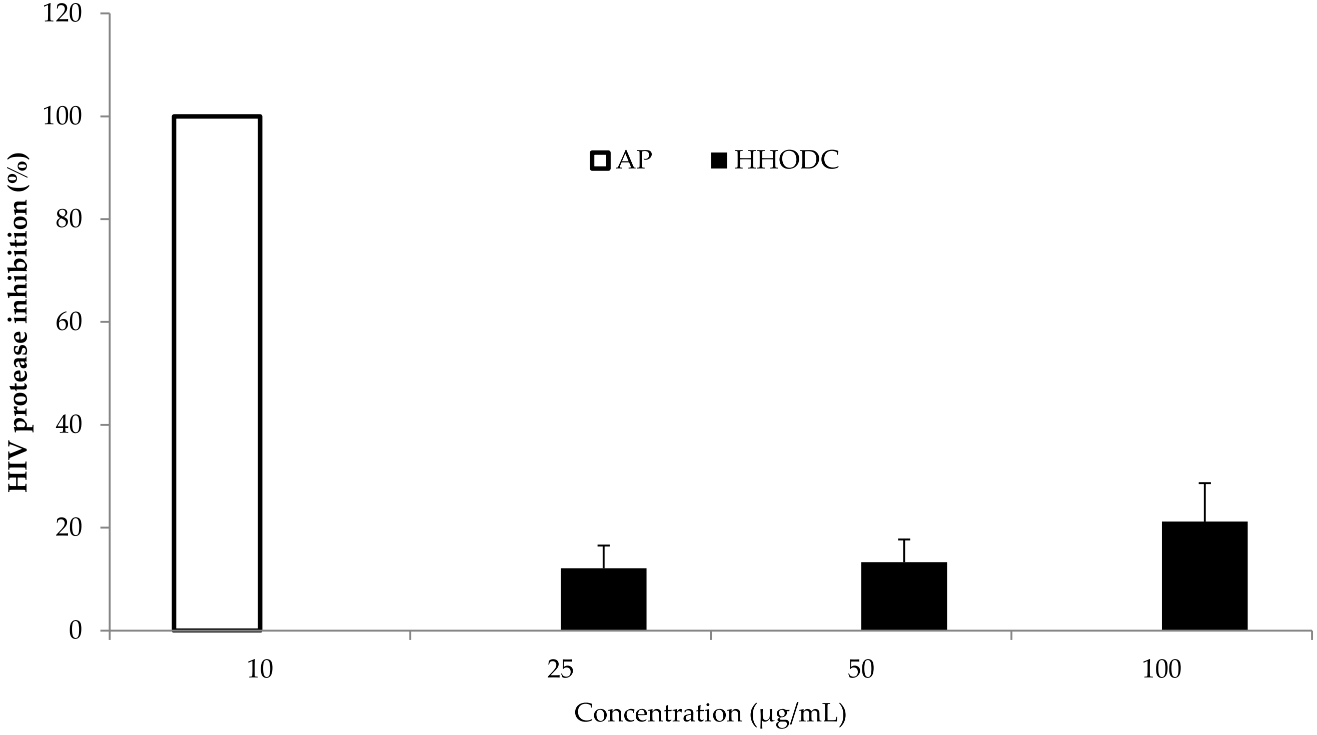
120
100
80
60
40
20
0
HIV protease inhibition (%)
AP
HHODC
10
25
50
100
Concentration (µg/mL)

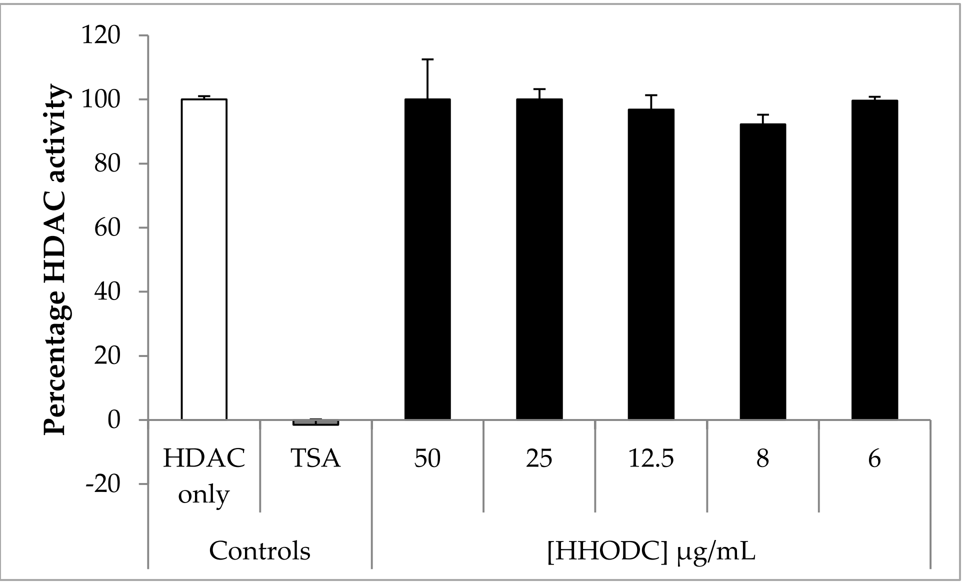

Percentage HDAC activity
120
100
80
60
40
20
0
-20
HDAC only
TSA
50
25
12.5
8
6
Controls
[HHODC] µg/mL

(a)

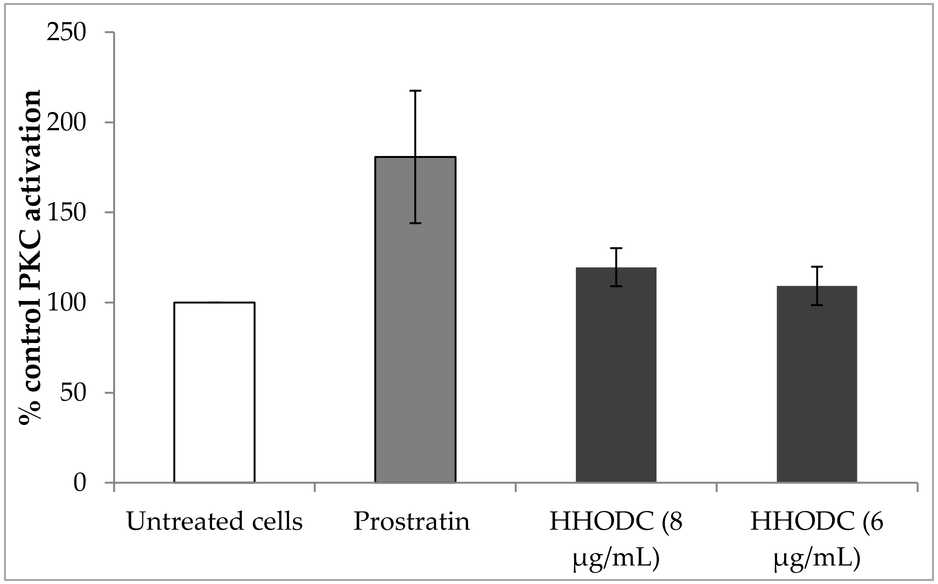

% control PKC activation
250
200
150
100
50
0
Untreated cells
Prostratin
HHODC (8 µg/mL)
HHODC (6 µg/mL)

(b)

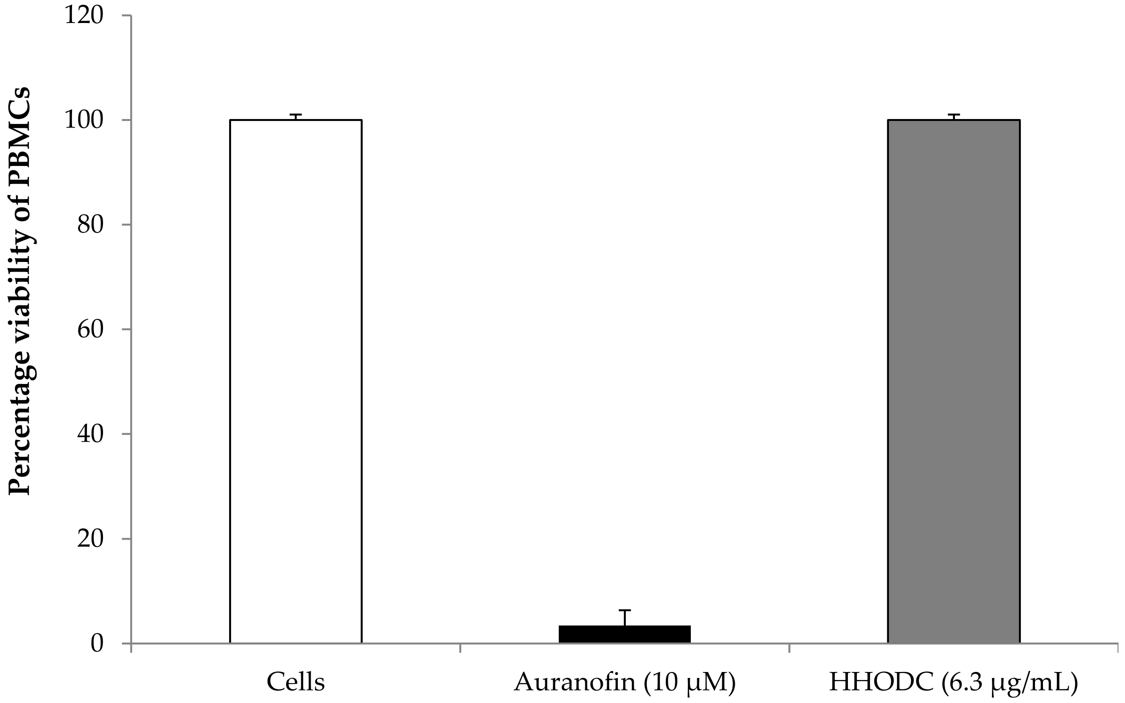

Percentage viability of PBMCs
120
100
80
60
40
20
0
Cells
Auranofin (10 µM)
HHODC (6.3 µg/mL)
